# Engineering benefits of replacing natural sand with manufactured sand in landfill construction

**DOI:** 10.1038/s41598-023-32835-7

**Published:** 2023-04-20

**Authors:** Anjali G. Pillai, Madhavi Latha Gali

**Affiliations:** Department of Civil Engineering, Indian Institute of Science, Bangalore, India

**Keywords:** Engineering, Materials science

## Abstract

Translational sliding failures in landfills are often triggered by inadequate shear strength of interfaces in liners and covers. Geosynthetic Clay Liners (GCL) are used in different components of landfills to contain the leachate. GCLs are usually placed above a compacted sand subgrade to develop higher shear resistance. In the context of depleting natural sand resources, the present study explores the feasibility of replacing natural sand with manufactured sand (Msand) in landfill construction. Interface shear tests were performed on GCL in contact with river sand and Msand of similar gradation to evaluate the shear strength at different normal stresses and hydration conditions. It is found that Msand provides higher interface shear strength with GCL compared to river sand. Digital image analysis of tested specimens of GCL showed that variation in particle morphology of the two sands has direct influence on the microlevel interaction mechanisms governing the shear strength. Quantification of morphological parameters showed that Msand particles are angular and rough compared to natural sand particles, leading to higher particle interlocking. Hydration of the GCL reduced the interface shear strength, the effect being less in case of Msand. The study highlights that replacement of natural sand with Msand has added benefits.

## Introduction

**Figure 1:**
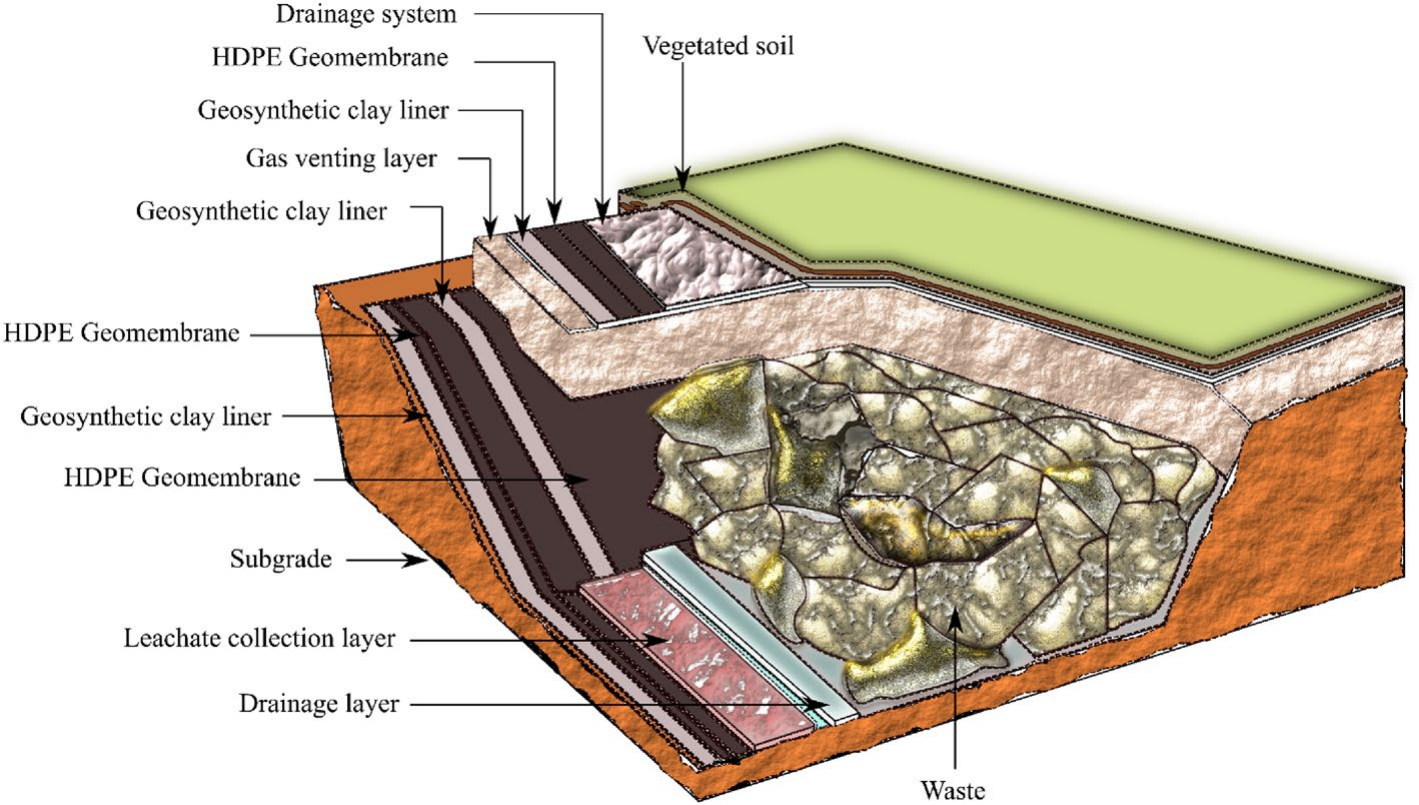
Schematic diagram of an engineered landfill.

Geosynthetic clay liners (GCL) are polymeric geocomposites that are used to contain environmentally harmful elements such as leachates in engineered landfills to prevent them from entering the ground and eventually contaminating the groundwater. GCLs comprise of bentonite clay in combination with polymeric materials like geomembranes and geotextiles. Bentonite is either adhesively bonded to geomembrane or encapsulated between two geotextiles, which are needle punched or stitch-bonded. GCLs are ideal replacement for conventional compacted clay liners (CCL) on account of their effective hydraulic properties, self- healing ability, cost effectiveness and easy installation benefits^1–3^. GCLs have several advantages over CCLs in terms of quality assurance, reduced thickness of layers, durability to freeze and thaw, easy accessibility and improved construction speed^4,5^. GCLs with woven or nonwoven geotextiles are commonly used to form interfaces with other geosynthetics and subgrade material. The placement of GCLs in liners and cover systems is shown in Fig. 1, in which GCLs are in contact with sand layers at various locations. The inhomogeneity in the lining and covers of landfills results in failures under normal stresses and shear stresses imposed by waste dumping and other special conditions like earthquakes. The primary cause of failure in the liners with GCLs is the translational sliding failure due to the insufficient shear strength at the GCL-sand interfaces, the chances being more in case of sloping grounds. Precise evaluation of interface shear strength of GCLs is required to control the sliding and other mechanical instabilities of landfills.

Literature on different interface tests performed using conventional direct shear box reveals that the development of friction and adhesion between the interacting layers is governed by several micro level interactions^6–9^. The advancement in technology has facilitated the investigation of interacting mechanism that affects the shear behaviour of interfaces at microlevel. Researchers have explored the effect of size and shape of sand particles on the interface behaviour with different types of reinforcements. The size of sand grains combined with roughness characteristics of the reinforcement controls the interface shear strength^10–13^. During their functioning in landfill, GCLs get hydrated due to their exposure to leachate or infiltrating precipitation, causing the swelling of encapsulated bentonite layer. Swelling of bentonite can considerably reduce the interface shear strength. Extrusion and lateral swelling of bentonite depend on the surface texture of the GCL^14–16^.

Extraction of natural sand for numerous constructional activities has significant impact on river and marine ecosystems, fluctuations in groundwater table and reduction in sediment supply. Though natural sand is considered as an appropriate subgrade material to be used in conjunction with GCLs to derive good interface shear strength, its non-availability ushers the researchers to investigate alternative subgrade materials. The suitable substitute must lower the negative impact on the environment and give a more sustainable and economical solution. Manufactured sand, popularly known as Msand is artificial sand obtained by crushing of stones to produce sand-sized particles. Several studies have investigated the strength properties and effectiveness of using Msand in manufacturing of concrete instead of river sand. Literature suggests that Msand provides better connection of cementitious matrix than river sand, due to the favourable surface characteristics of Msand particles^17–19^. Limited research has explored the use of Msand for applications other than concrete manufacturing.

The present study contributes to the feasibility of utilizing Msand as a suitable replacement for river sand to provide a superior GCL-Msand interface to be used in liners of landfill. This is highlighted through the comparative interface shear tests on both GCL-River sand and GCL-Msand interfaces along with computation of shear strength parameters. Further, the effect of GCL hydration on shear strength parameters is understood for completely saturated condition of subgrade material. The results are corroborated with image-based studies of particle shape and microlevel changes to the tested GCL surfaces. Morphological parameters of the sand, including the shape and size of the grains, greatly influence the micro-level interactions and in turn the shear strength of the interfaces. Studies carried out by earlier researchers on polymeric surfaces interfacing with angular sand grains and spherical glass beads pointed out the difference in interface shear strength with change in particle shape^20,21^. The current study explores these aspects in detail and proposes using Msand in the construction of liners and cover systems of landfill on account of its improved interface shear strength with GCLs, reduced hydration effects, and long-term sustainability benefits.

## Test materials

### Geosynthetic clay liner (GCL)

**Figure 2:**
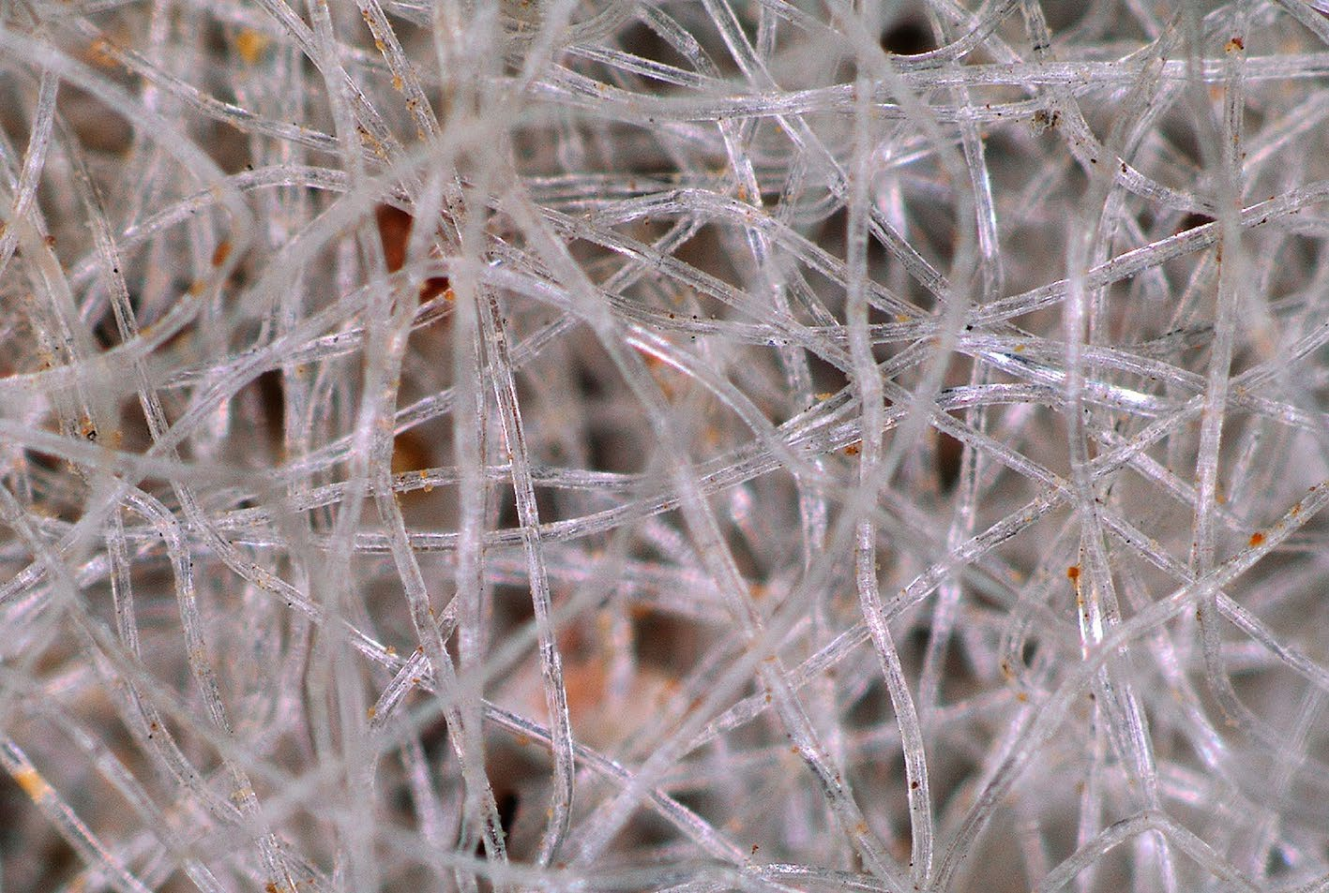
Microscopic image of nonwoven surface of virgin GCL at 32× magnification.

The GCL used in this study is Macline GCL-W, which has a layer of dry sodium bentonite encapsulated between a thermally bonded nonwoven geotextile and a filament woven geotextile. The bentonite clay with 70% montmorillonite has 650% water absorption capacity and free swell capacity of 12 ml/g. Literature suggests that nonwoven side of GCL has better interface shear strength due to its randomly oriented fibres^22–24^ and hence the same is adopted for shear tests in this study. Figure 2 shows the image of the nonwoven side of the virgin GCL taken at 32× magnification using a stereo microscope (SZX10), indicating a multi-layer web-like structure of the geotextile.

### Sands

**Figure 3:**
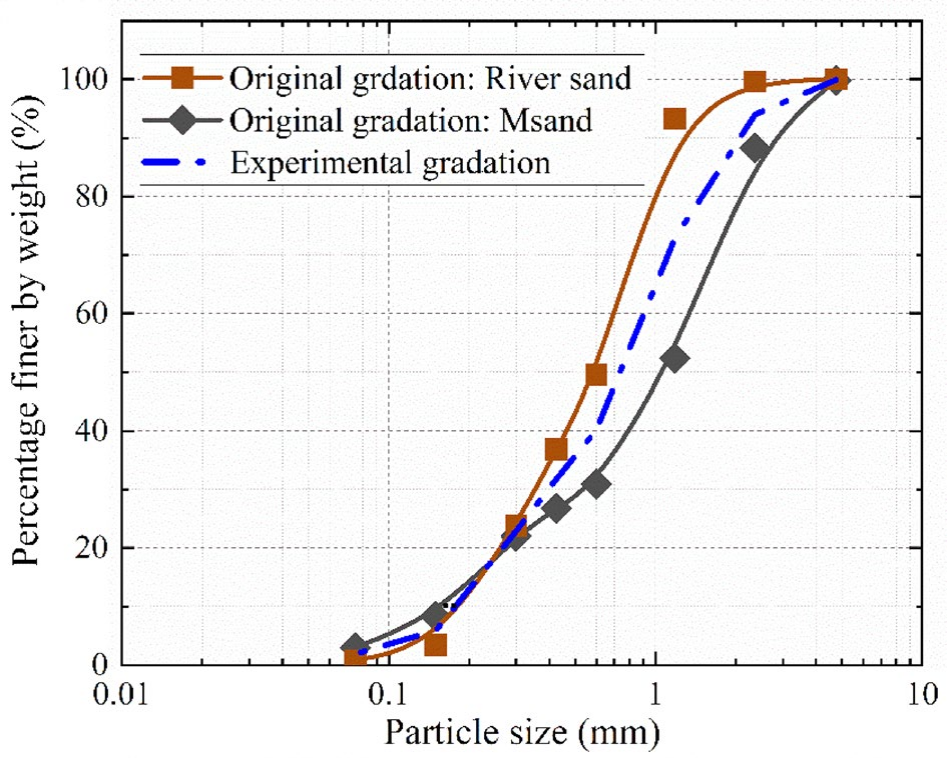
Grain size distribution of sands and experimental gradation.

**Figure 4:**
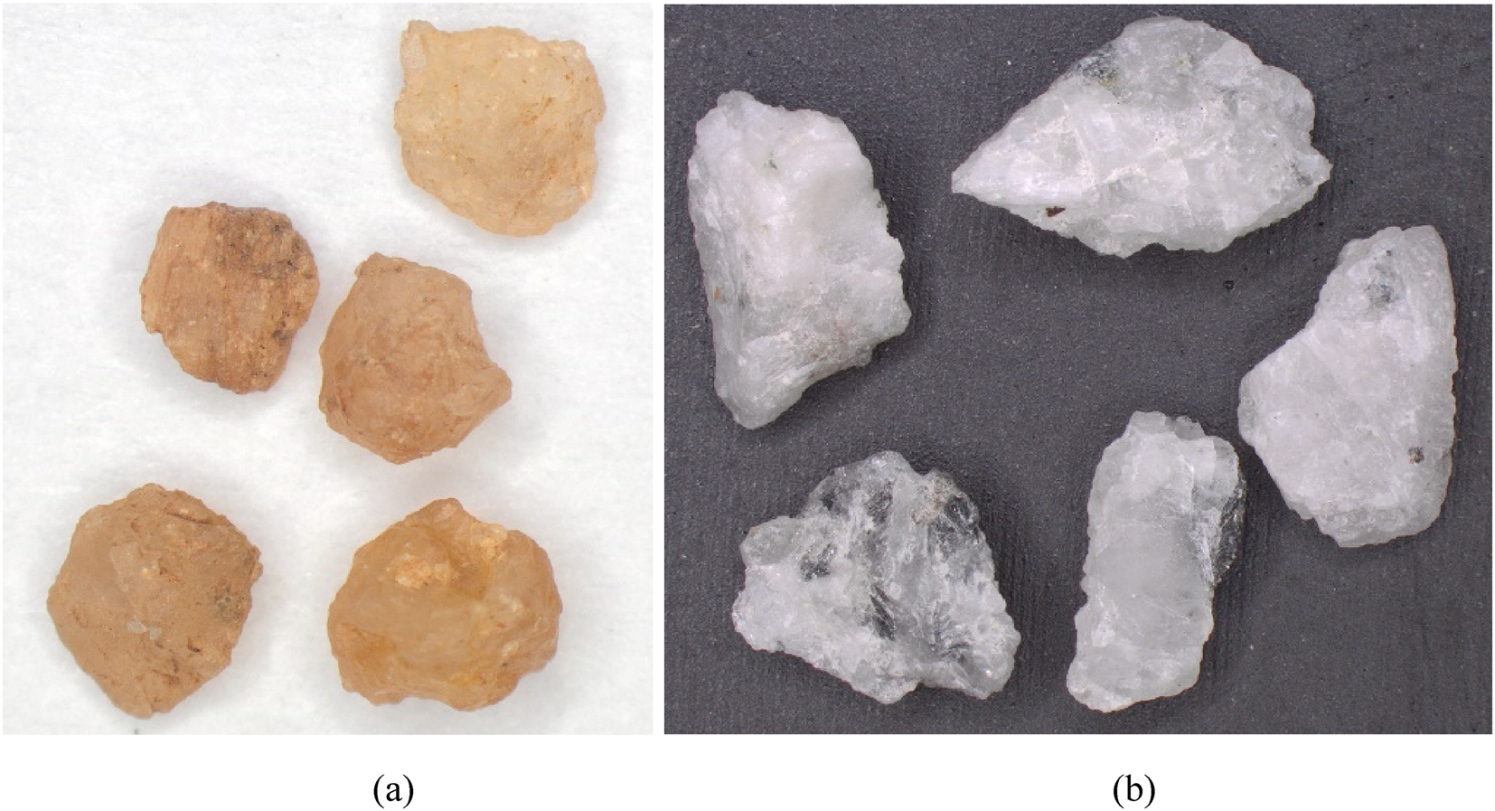
Microscopic images of typical sand particles at 25× magnification (**a**) river sand and (**b**) manufactured sand.

Sieve analysis was carried out on natural river sand and manufactured sand, as per ASTM C136 (2014). Based on the particle size distribution shown in Fig. 3, the river sand and Msand, in their natural gradation, are classified as poorly graded sand (SP) and well graded sand (SW), respectively, according to United Soil Classification System (USCS). To eliminate the effects of grain size and focus on the grain shape effects, a common experimental gradation for river sand and manufactured sand (Msand) is used in the present study. as shown in Fig. 3. The chosen gradation of sands was obtained by proportioning and procuring required quantity from specific size fractions of both sands so that both sands attain the gradation that is classified as poorly graded sand (SP). The chosen gradation of sands has identical grain sizes by weight fractions and the shape of the grains remain as per their natural state. Figure 4 shows the images of river sand and manufactured sand of 0.6 mm size. Table 1 lists the gradation parameters and physical properties of sands in their experimental gradation. The maximum void ratio was obtained from vibratory table test as per ASTM D4254 (2016), minimum void ratio was obtained as per ASTM D4253 (2019). Based on direct shear tests conducted as per ASTM 3080-04 (2012), the internal friction angles were obtained as 44º and 41º, respectively, for the chosen gradation of manufactured sand and river sand, at 80% relative density.

**Table 1:** Physical properties of sand.

Property	River sand	Manufactured sand (Msand)
USCS classification	Poorly graded (SP)	Poorly graded (SP)
Coefficient of uniformity, *C*_*u*_	5.82	5.82
Coefficient of curvature, *C*_*c*_	1.11	1.11
Specific gravity (*G*)	2.69	2.57
Maximum void ratio (*e*_*max*_)	0.54	0.54
Minimum void ratio (*e*_*min*_)	0.47	0.46
Angle of internal friction (*ϕ*_sand, dry_)	41º	44º
Angle of internal friction (*ϕ*_sand, saturated_)	34º	37º

## Experimental methodology

**Figure 5:**
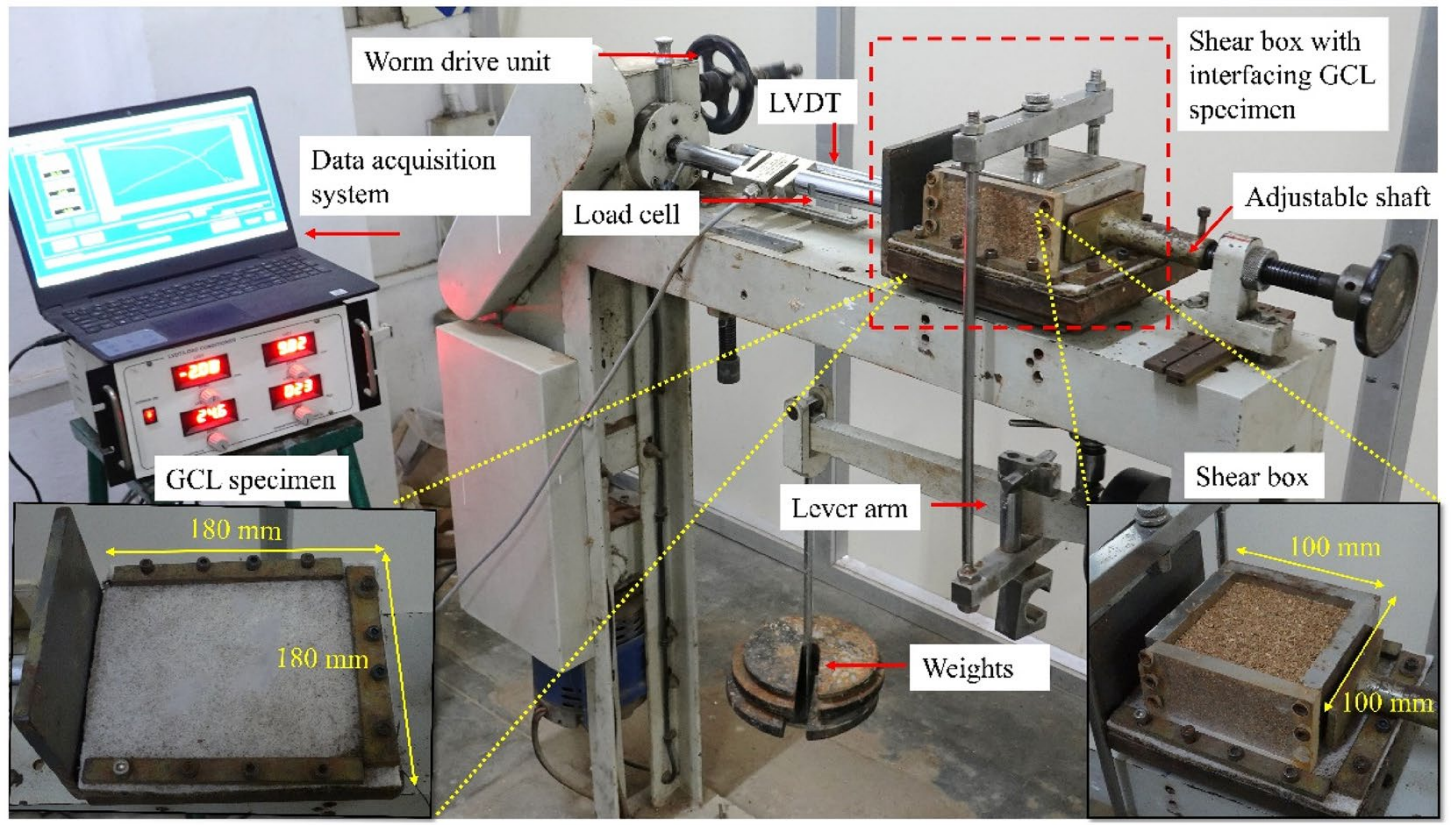
Interface direct shear setup.

All interface shear tests were performed using the direct shear test setup modified by Vangla and Latha^25,26^. This setup replaces the bottom shear box of conventional direct shear test setup with a movable rigid square steel plate of 180 mm sides to fix the geosynthetic materials. In the current study, GCL specimens measuring 180 mm × 180 mm were cut from the roll and fixed to the steel base using gripper plates and screws. GCL was fixed to the steel plate in a way that its nonwoven geotextile surface is upwards. The upper shear box of 100 mm × 100 mm size was placed on top of the fixed GCL and filled with sand at a relative density of 80%. The maximum and minimum void ratios of sand reported in Table 1 were used for the computation of required weight of sand to achieve the specific relative density. Sand was filled in three separate layers, each individual layer hand-compacted to achieve one-third of the total 50 mm height of the specimen. After a normal load is applied through the lever-arm mechanism, the steel base was moved horizontally on rollers, causing shear along the interface of the GCL and sand. The digital data acquisition system records the applied shear force through a horizontal load cell and corresponding horizontal movement of the base using an LVDT (linear variable differential transformer), respectively. Figure 5 shows the details of the interface test setup used in the study.

Strain controlled interface shear tests were performed at normal stresses of 100 kPa, 60 kPa and 30 kPa and 7 kPa at a shear rate of 1.15 mm/min as per ASTM D6243 (2016). These normal stresses simulate the overburden stress in liners and capping of landfills of moderate height. Interface tests were carried out on GCL-River sand and GCL-Msand interfaces. Tests were carried out on GCLs interfacing with dry and saturated sand. Saturation of sand in experiments represents the worst-case field scenario in which fluctuating groundwater levels and infiltration of precipitation cause saturation of sand subgrade. The minimum saturation water content of sand was computed as 18% from specific gravity and void ratio relationships and the same was used in tests with saturated sand.

## Results and discussions

### Assessment of shear strength under dry and hydrated conditions

**Figure 6:**
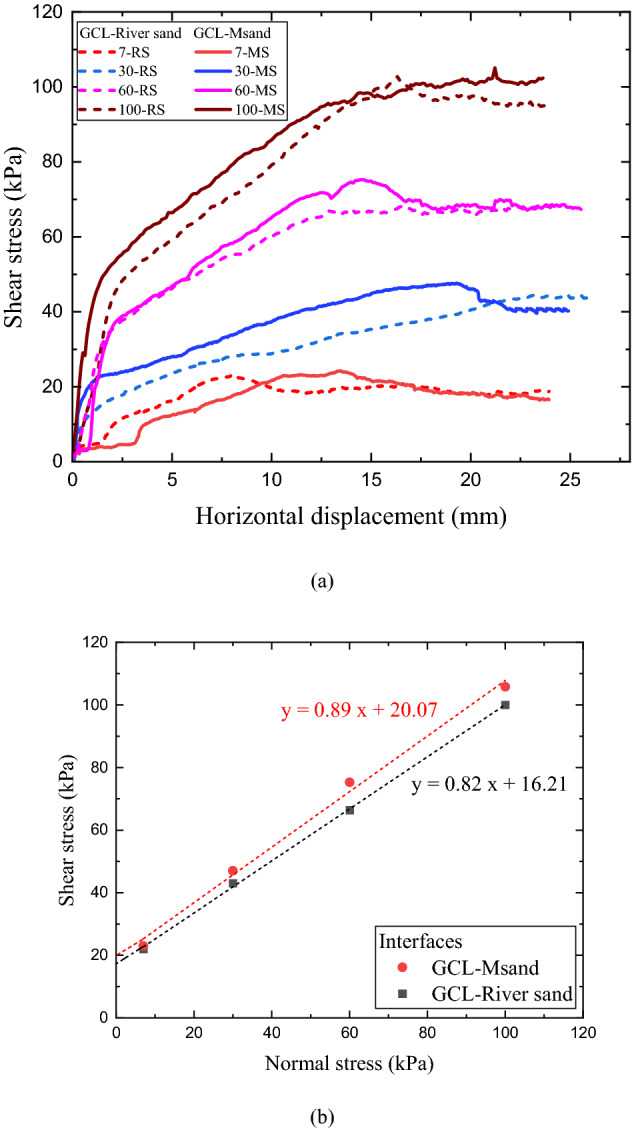
Shear response of GCL- sand interfaces under dry condition (**a**) stress–displacement response, (**b**) failure envelopes.

The interface shear tests were designed to understand the effects of overburden stress levels represented through normal stress variations, grain shape effects through natural and Msand having significant difference in their particle shape and hydration effects through dry and wet tests. The shear response of GCL-River sand and GCL- Msand interfaces in dry condition at different normal stresses is presented in Fig. 6. Variation of shear stress with displacement is shown in Fig. 6a and Mohr–Coulomb failure envelopes are shown in Fig. 6b. Most of these initial tests were repeated to confirm the reproducibility of results. For both GCL-River sand and GCL-Msand interfaces, peak shear stress increased with the increase in normal stress, which is indicative of increased interlocking mechanism between the fiber of geotextile and sand particles under enhanced confinement effect of overburden. Further, the plots show that higher peak shear stress is attained for GCL-Msand compared to GCL-River sand interfaces. Since the test conditions and gradation are maintained identical, the difference in the shear behaviour can only be related to the shape of the sand particles. The internal reinforcing fiber of GCL resist the applied shear force, contributing to the overall shear strength. They transmit the shear force from upper layer to the lower layer of GCL. The post-peak reduction in shear stress can be linked to the extension of reinforcing fiber at large shear strains, which causes loss of tensile strength, leading to the reduction in the interface shear strength^11–13^. The values of interface friction angle (*δ)* and interface adhesion (*a*_*p*_) for dry GCL-sand interfaces computed from the best-fit lines of the failure envelopes considering peak interface shear stresses at different normal stresses shown in Fig. 6b are listed in Table 2. All GCL-sand interfaces showed lesser friction angles compared to sand-sand shear tests. Friction efficiency of GCL-sand interfaces, which is defined as *δ*/*ϕ*, is always less than 1.0, as shown in Table 2.

**Table 2:** Shear strength parameters from shear tests on GCL-sand interfaces.

Interface	Water content in sand (*w*) in percentage	Interface friction angle *δ* (degrees)	Interface adhesion *a*_*p*_ (kPa)	Friction efficiency of the interface *δ/ϕ*_sand_
GCL-Msand (dry)	0	41.66	20.07	0.94
GCL-River sand (dry)	0	38.35	16.21	0.93
GCL-Msand (saturated)	18	32.21	10.23	0.87
GCL-River sand (saturated)	18	28.81	9.57	0.84

**Figure 7:**
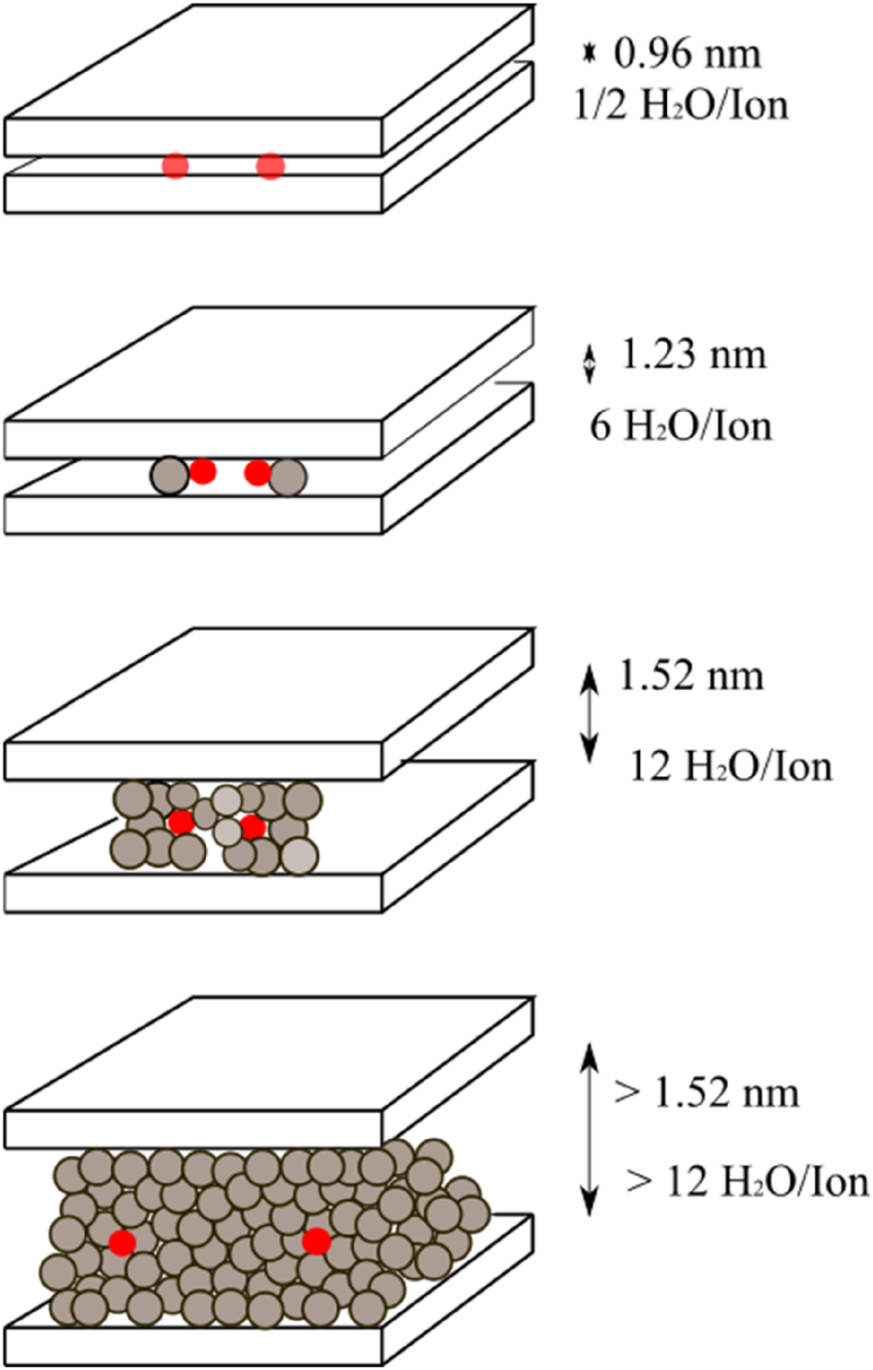
Crystalline swelling mechanism of clay minerals by hydration.

**Figure 8:**
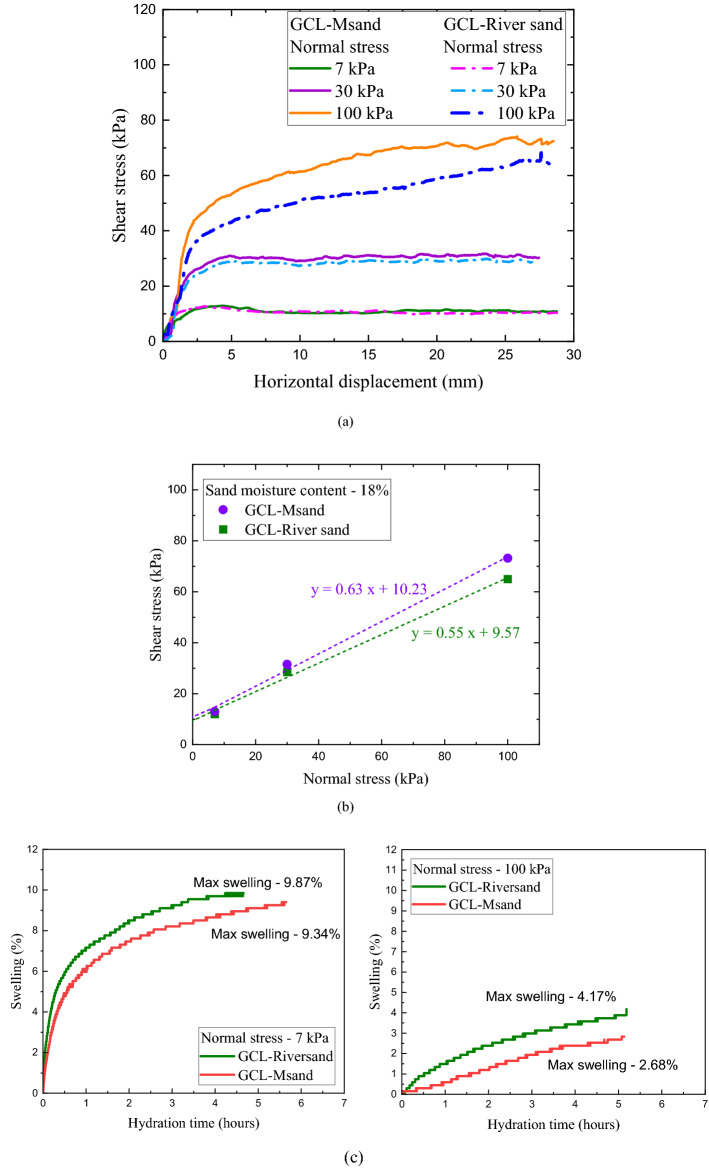
Shear response of GCL-sand interfaces under saturated condition (**a**) stress–displacement response, (**b**) failure envelopes, (**c**) swelling-hydration time response.

Bentonite improves the hydraulic performance of GCL, giving it the ability to self-heal. Upon hydration, bentonite increases its volume by 600%, which creates significant impact on the shear strength of GCL. Figure 7 shows the crystalline swelling mechanism of bentonite as explained by Ruedrich et al.^27^. In field, suction of moisture from the interfacing subgrade can lead to bentonite hydration in GCL. The fluctuating groundwater level and infiltrating rainwater can result in abrupt rise of water content of subgrade, thereby negatively impacting the shear strength of GCL-sand interfaces. The worst-case scenario for this reduction of interface shear strength would be the completely saturated condition of the sand subgrade. In this study, interface tests under saturated conditions were conducted to examine the impact of bentonite hydration on the computed interface shear strength parameters. The water content in sand was maintained at 18% in these tests to achieve complete saturation and the GCL specimens were hydrated by suction of moisture from the sand. The normal stresses used for this set of tests were 7 kPa, 30 kPa and 100 kPa. The low normal stress of 7 kPa was used to permit high swelling of bentonite and high normal stress of 100 kPa was used to facilitate the extrusion of bentonite on to the surface of GCL. Both these phenomena influence the interface shear behaviour of GCL-sand interfaces. The stress- displacement response of GCL-River sand and GCL-Msand under saturated subgrade condition are shown in Fig. 8. A significant difference in peak shear stress of river sand and Msand interfaces was observed at higher normal stress of 100 kPa, as seen from Fig. 8a. When Fig. 6a for dry tests and Fig. 8a for saturated tests are compared, significant reduction in shear stress at all normal stresses was observed under saturated conditions. The hydration of GCL resulted in the swelling of bentonite, exerting tensile forces on the reinforcing fibers, and thereby impacting the interface shear strength. At higher normal stress, swelling of bentonite is opposed by the overburden stress, leading to the bentonite extrusion onto the interface through the voids of the nonwoven geotextile surface. Figure 8c shows the swelling of GCL specimens with the hydration time for normal stresses of 7 kPa and 100 kPa. As stated earlier, higher swelling is observed in interfaces tested under 7 kPa, which signifies the higher volumetric expansion of GCL upon hydration as compared to interfaces tested at a normal stress of 100 kPa. At higher normal stress, the swelling is restricted and found to be 5–7% lower. The volumetric change is observed to be more for GCLs interfaced with river sand. Bentonite is extruded through the voids of the nonwoven surface of the GCL and forms a slimy layer at interface. The extruded bentonite along with the lubricating layer of water at interface, reduces the frictional resistance. Computation of interface shear strength for saturated conditions through Mohr- Coulomb failure envelopes is shown in Fig. 8b and the values of interface friction angle (*δ*) and interface adhesion (*a*_*p*_) are listed in Table 2. As observed, the adhesion and friction angles of the saturated interfaces are significantly lower than those of the dry interfaces. The interface friction angle was reduced by about 10° from dry to saturated condition, both for river sand and Msand interfaces and the interface adhesion was reduced by 7–10 kPa from dry to saturated condition, for the reasons explained above. The reason for the reduction in frictional resistance with saturation is the slimy layer of extruded bentonite under saturated conditions and the interaction of sand particles with this layer, which restricts the efficient sand-fiber interlocking. The lubricating layer of water also reduces the frictional resistance at the GCL-sand interfaces under saturated conditions. Even though identical gradation was maintained for river sand and Msand in this study, Msand interfaces showed significantly higher friction angle and adhesion compared to river sand interfaces in all conditions due to particle morphology effects, which are explained in subsequent sections.

### Particle morphology using image analysis

The influence of sand grain shape on the shear response of GCL-sand interfaces is evident from the analysis of the test results. The availability of high-end imaging techniques and robust computational tools have made accurate quantification of particle shape possible. By using imaging techniques, soil-geosynthetic interaction mechanisms can be accurately analysed and the same can be correlated to the measured mechanical response to gain deeper insights. In this study, digital imaging techniques were employed to differentiate and quantify the shape parameters of sand grains and to understand the microlevel changes to the tested GCL surfaces to explain the interaction mechanisms at the interface.

**Figure 9:**
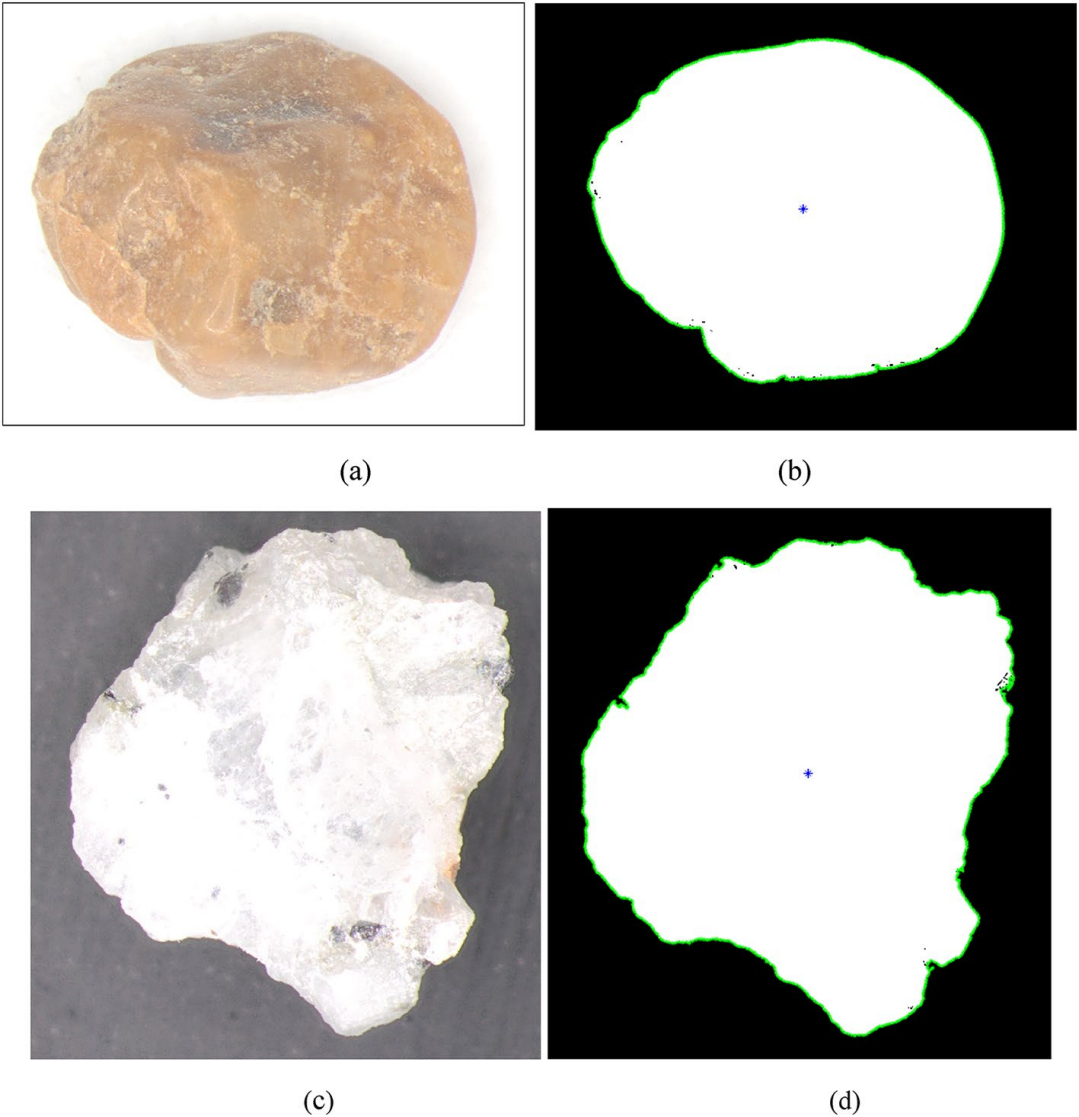
Microscopic and binary images of typical sand particles (**a**) Microscopic image of river sand particle (**b**) Binary image of river sand particle (**c**) Microscopic image of Msand particle (b) Binary image of Msand particle.

**Figure 10:**
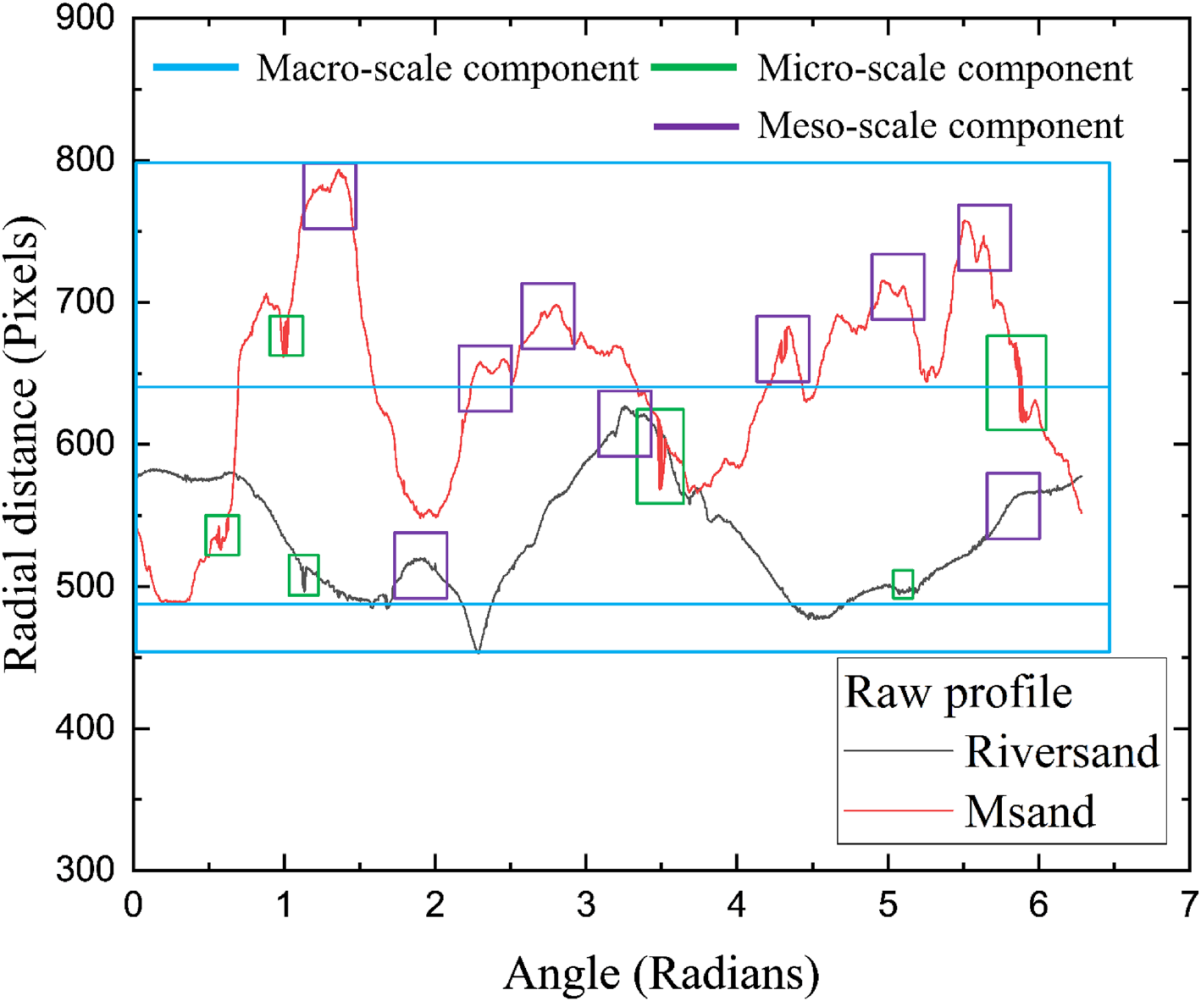
Raw profiles of typical river sand and Msand particles with multiscale shape components marked.

The grain shape comprises of three multiscale components—form (macro-scale), roundness (meso-scale) and surface texture (micro-scale)^28^. The *form*, a macro-scale component, describes the deviations in particle proportions. The meso-scale component*, roundness*, describes the undulations or corners along the particle outline. The surface texture, micro-scale component, defines the minute roughness characteristics on the particle surface. Several shape parameters were defined in literature to characterize the particle shape using particle images and computational techniques. The most widely accepted shape parameters are sphericity, roundness and roughness given by Wadell^29,30^, which were widely used by many subsequent researchers^13,25^. Sphericity representing the closeness of the grain shape to a sphere, roundness representing the smoothness of the grain boundary and roughness representing the micro-scale irregularities on the grain boundary, are collectively used to represent the overall grain shape. In this study, an algorithm is written in MATLAB to quantify Wadell’s shape parameters of sand grains. For this purpose, microscopic images of sand particles were converted into binary images through image segmentation in MATLAB and shape parameter quantifications were carried out on the binary images. Figure 9 shows the microscopic and binary images of typical grains of river sand and Msand, both of 0.6 mm size. Figure 9b,d show the outline of the grain along with the centroid, for river sand and Msand grains, respectively. These grain outlines are plotted in the spatial domain of grain radius in pixels and angle in radians, to obtain the raw profile of the individual sand particles, as shown in Fig. 10. The raw profile consists of the three multiscale features of the grain, which are form, roundness, and roughness, which are identified and marked for both the sand particles in Fig. 10. While the macro-scale component encompasses the complete raw profile, the meso-scale component corresponds to the major peaks and troughs of the raw profile and the micro-scale component corresponds to the closely spaces clusters of minute deviations in the profile. The raw profile of the Msand particle shows more meso-scale and micro-scale components of shape, indicating the angularity and rough texture of Msand particle compared to the river sand particle. Further shape quantifications were carried out on the binary images of 200 individual particles in different size fractions for both the sands using MATLAB algorithm^29^ and the average values of shape parameters were computed. The average sphericity, roundness and roughness were obtained as 0.78, 0.38 and 0.0024, respectively for Msand and 0.84, 0.42 and 0.001, respectively for river sand^31–33^. The natural weathering and erosion processes responsible for the formation of river sand particles gave them higher sphericity and roundness compared to Msand particles which were stone-quarried. The average roughness value of the Msand particles is twice the average roughness of the river sand because of the mechanical process involved in crushing of rocks to manufacture the Msand.

### Interaction mechanisms of GCL-sand interfaces

**Figure 11:**
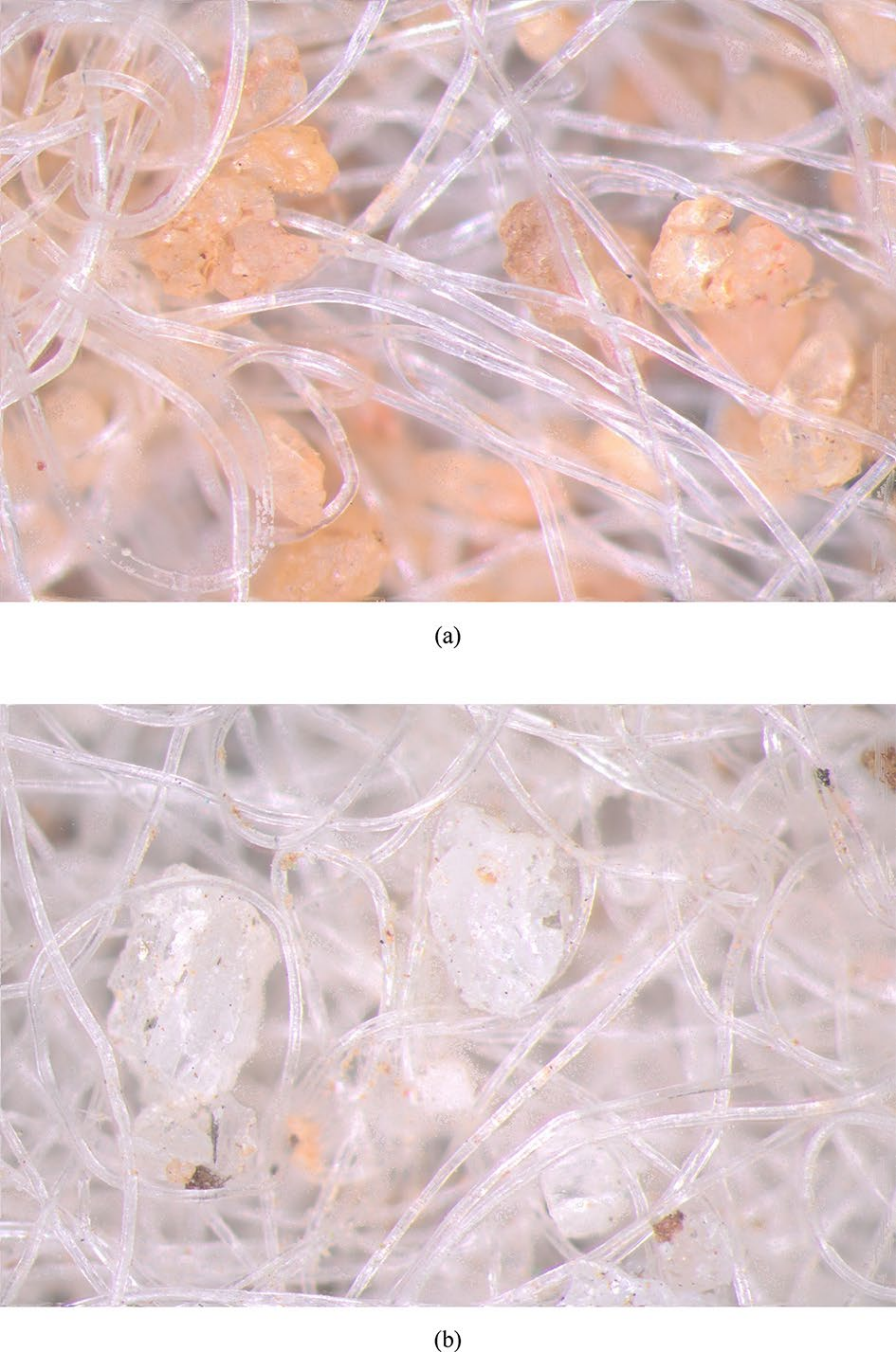
Images of tested surfaces of GCL showing particle-fibre interlocking (**a**) GCL-River sand (**b**) GCL-Msand.

The sharper elongated and rougher Msand particles generate higher friction upon interaction with other surfaces such as GCL as compared to river sand particles, which is confirmed from the results of interface shear tests. While the sands are being sheared on GCL, apart from the adhesion and friction between sand particles and GCL, there is another important mechanism that significantly contributes to the shear strength of GCL-sand interfaces, which is the sand-fiber interlocking. Through Fig. 11, sand particle interlocking within the fibers of GCL can be clearly visualized for GCL-River sand and GCL-Msand interfaces. Using binary image segmentation and *region properties* function in MATLAB, fibers and particles were differentiated and the percentage area of sand particle entrapment on GCL surfaces was computed. Under dry conditions, the area of entrapment of sand particles for GCL-River sand and GCL-Msand interfaces was 3.44% and 2.29%, respectively at a normal stress of 100 kPa. Without other influences, increase in particle entrapment must result in increase in the interface shear strength. However, GCL-Msand interfaces showed higher shear strength compared to GCL-river sand interfaces despite the relatively lesser entrapment. The reason for this higher shear strength is the shape of the Msand particles, which compensated for all other effects.

**Figure 12:**
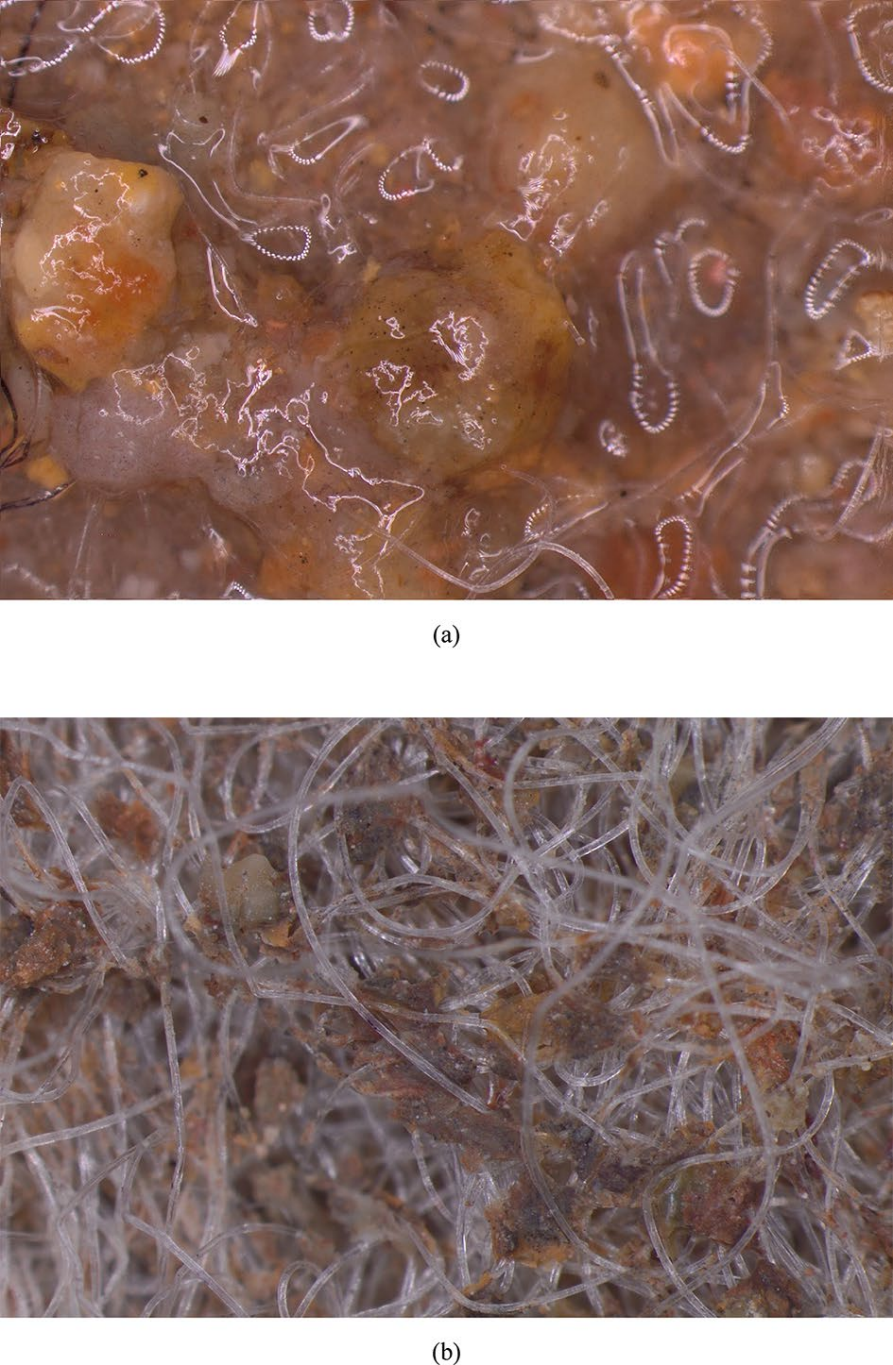
Images of tested GCL surface after saturated shear tests taken at 20× magnification (**a**) bentonite extrusion under hydration, (**b**) extruded bentonite after drying up the GCL.

In saturated tests, swelling and extrusion of bentonite greatly influenced the interface shear strength as well as the particle entrapment. Figure 12a shows the tested surface of GCL after a saturated test in which extruded bentonite along with a lubricating film of water can be clearly observed. The extruded bentonite forms a slimy sticky layer at the interface, which reduces the friction at the interface. The slimy bentonite layer sticking to the fibers can be seen in Fig. 12b, which is the photograph of the GCL dried after a saturated test. This layer causes higher sand particle entrapment because of its stickiness. The area of entrapped sand particles after saturated tests was higher and computed as 35.55% in GCL- River sand and 20.80% in GCL-Msand interfaces, at a normal stress of 100 kPa. These results prove that bentonite hydration effects are more in river sand. Shear strength of GCL-Msand interfaces is higher compared to river sand particles even under hydrated conditions, because of the particle shape effects.

The interface shear tests, and image analyses carried out in this study bring out the benefits of replacing river sand in liners and capping systems of landfills with Msand and provide scientific explanations for the same. The practical benefits of this study lie in the reduced usage of natural sand in landfill construction, which has long-term environmental benefits. Cost of manufactured sand is much less compared to the cost of river sand, and hence the replacement has high economic benefits. Feasibility of production of specific gradation of Msand to derive maximum benefits in terms of interface shear strength is an added advantage. Findings from the present study can be used to derive empirical relations between the shape parameters of sand and the interface shear strength with GCLs using multivariable regression analysis. However, such relations will be more meaningful if the data includes tests with different GCLs and different water contents in the sand, which can be investigated in future.

## Conclusions

Shear strength of GCL-sand interfaces and its dependency on grain shape are investigated in this study through shear tests on GCL interfacing with river sand and manufactured sand (Msand) under dry and saturated conditions. Micro level shearing and interlocking mechanisms and the effects of grain shape on these mechanisms are studied through image analysis. Major conclusions of the study are listed below.

1. Higher shear strength was observed for GCL-Msand interfaces compared to GCL-river sand interfaces because of the elongated shape and rough surface texture of the Msand particles. Both interface friction angle and adhesion are relatively higher for GCL-Msand interfaces.
2. Apart from friction and adhesion, sand-fiber interlocking at the interface significantly contributes to the shear strength of GCL-sand interfaces. Effective interlocking was observed in case of Msand compared to river sand by virtue of grain shape, leading to higher frictional contact with fibers.
3. In saturated tests, bentonite was swollen upon hydration and got extruded onto the interface, as visualized from the image analysis of GCL surfaces after the tests. Bentonite extrusion resulted in significant loss of shear strength at the interface, the loss being lesser in case of Msand since particle shape effects compensated for the strength loss.
4. This study proves that Msand can be an economical, sustainable, and effective replacement for river sand as a subgrade material in the construction of engineered landfills.

## Data Availability

The datasets used and/or analysed during the current study available from the corresponding author on reasonable request.
